# The MA Helix Is Important for Receptor Assembly and Function in the α4β2 nACh Receptor

**DOI:** 10.3390/membranes13120891

**Published:** 2023-11-29

**Authors:** Dorottya I. Fricska, Susanne M. Mesoy, Sarah C. R. Lummis

**Affiliations:** Department of Biochemistry, University of Cambridge, Tennis Court Road, Cambridge CB2 1QW, UK; dif22@cantab.ac.uk (D.I.F.); susanne.mesoy@chem.ox.ac.uk (S.M.M.)

**Keywords:** neurotransmitter receptor, Cys-loop receptor, acetylcholine receptor, pentameric ligand-gated ion channel, intracellular domain

## Abstract

Pentameric ligand-gated ion channels (pLGICs) are expressed throughout the central and peripheral nervous systems of vertebrates and modulate many aspects of human health and disease. Recent structural and computational data indicate that cation-selective pLGICs contain a long helical extension (MA) of one of the transmembrane helices. The MA helix has been shown to affect both the membrane expression of, and ion conductance levels through, these pLGICs. Here we probe the functional effects of 68 mutations in the MA region of the α4β2 nicotinic acetylcholine receptor (nAChR), using a voltage-sensitive membrane dye and radioligand binding to measure receptor function and expression/assembly. We found seven alanine mutations in a stretch of the MA helix that prevent correct receptor folding and/or assembly, as evidenced by the lack of both function and ligand binding. A further two alanine mutations resulted in receptors that were capable of binding ligand but showed no functional response, and we propose that, in these mutants, ligand binding is insufficient to trigger channel opening. The data clarify the effect of the MA helix, and as the effects of some of our mutations in the α4β2 nAChR differ from the effects of equivalent mutations in other cation-selective pLGICs, we suggest that residues in the MA helix may play subtly different roles in different receptors.

## 1. Introduction

**Figure 1:**
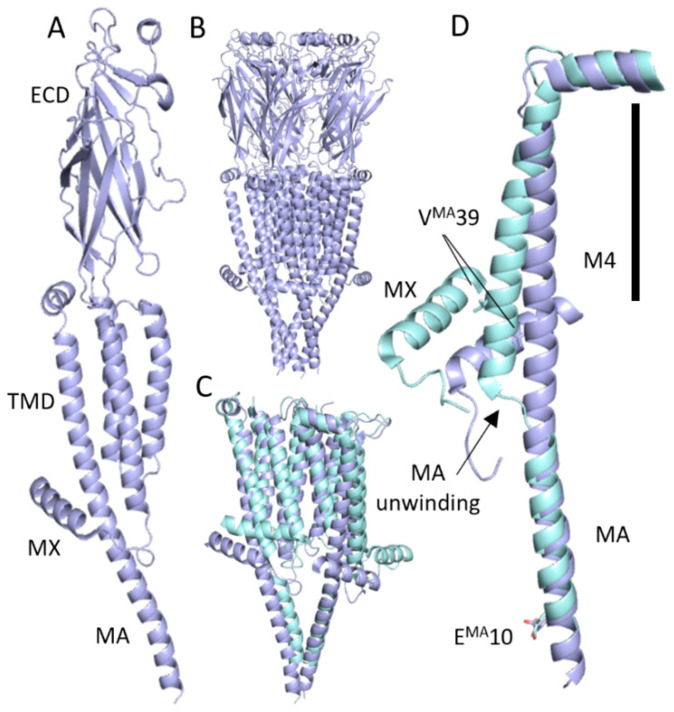
Structures of the α7 nAChR in the closed (dark blue, PDB 7KOO) and activated (light blue, 7KOX) states. (**A**) Single subunit showing major structural domains. (**B**) Receptor overview. (**C**) TMD and ICD of the open and closed states of two subunits. (**D**) M4, MX, and MA, showing unwinding of MA on receptor opening. The black line indicates the approximate location of the plasma membrane. Residue ^MA^39 is the most C-terminal residue investigated in this study, and residue ^MA^10 shows the relative positioning of the two helices below the membrane.

Pentameric ligand-gated ion channels (pLGICs) are neurotransmitter-gated ion channels that mediate fast synaptic transmission in the central nervous system, underpinning muscle action, gut activity, and a range of neurological functions. The archetypal pLGIC is the nicotinic acetylcholine receptor (nAChR), whose structure and function has been extensively studied over the last 50 years (e.g., [1,2,3,4,5]). The nAChR, like all pLGICs, consists of five subunits surrounding the ion channel pore. Neurotransmitter binding occurs in the extracellular domain (ECD), which is primarily β-sheet, while the α-helical transmembrane domain (TMD) is responsible for ion transduction [6,7]. In addition, vertebrate pLGICs contain a long (around 100–250 amino acids) intracellular domain (ICD) formed by the loop between transmembrane helices 3 and 4. This domain is often omitted from structural studies, but in some structures of cation-selective pLGICs, some parts have been elucidated; this includes two α-helices: the MX helix at the N-terminal end and the MA helix at the C-terminal end (Figure 1) [8]. 

Early structures revealed the presence of the MA helix in the ICD and its contribution to apertures close to the plasma membrane, named ‘lateral portals’ [9]. Since then, the MA helix has been shown to contribute both to membrane expression and single-channel conductance levels in cation-selective pLGICs, with recent structures showing significant helix unwinding (Figure 1) as the receptor moves from the closed to the open state [10,11,12,13]. 

**Figure 2:**
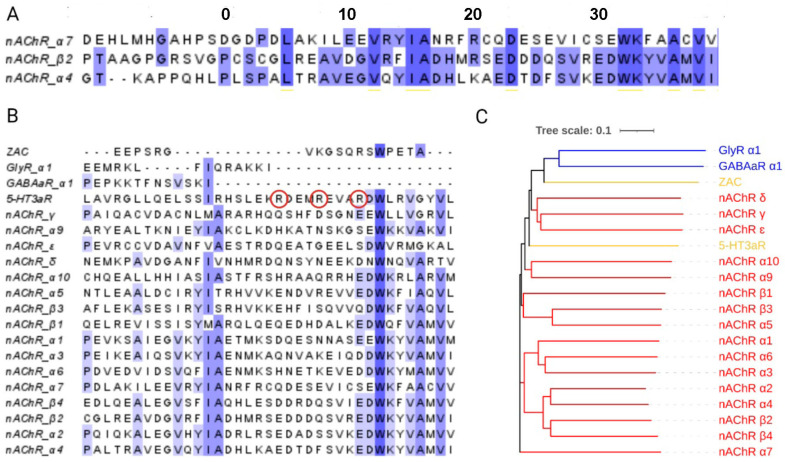
Comparison of pLGIC MA sequences. (**A**) A sequence alignment for 3 different nAChR subunits showing residue identity (~70% between α4 and β2 subunits) and the MA sequence numbering system used here. (**B**) A multiple-sequence alignment of MA helices from all nAChR subunits and representative subunits of other pLGICs reveals the most conserved residues. The three arginine residues that contribute to single-channel conductance in the 5-HT_3A_R subunit are indicated with red circles. (**C**) A phylogenetic analysis of the same sequences with nAChR subunits shown in red, other cationic subunits in yellow, and anionic subunits in blue.

Interestingly, sequence alignments indicate that anion-selective pLGICs may not have MA helices, as evidenced by the lack of sequence conservation after the M3 helix (Figure 2). This is in contrast to cation-selective pLGICs, which show some conservation of the MA region, and parallels the case of the MX helix at the other end of the ICD, which also shows sequence conservation in cation-selective, but not anion-selective, pLGICs [14]. Partial structures and AlphaFold structural predictions show the same pattern, consistently finding MA helices in most cation-selective pLGICs but not in anion-selective pLGICs [8,15,16,17].

In this work, we investigate the role of residues that make up the MA helix in the α4β2 nAChR and show that some residues are important for expression while others may play a role in allowing ion flux to occur in response to ligand binding. For ease of comparing MA helices between different receptors and subunits, we have instituted the numbering system shown in Figure 2, which starts at a largely conserved proline near the start of predicted MA helix, i.e., P562A (α4) and P417A (β2) in the nAChR are at position 0 and are referred to as P^MA^0A in the text and tables. The next residue after residue ^MA^39 is an aspartic acid that we count as the first residue of the M4 helix (D4.0, as described in [18]) due to its high level of conservation (likely due to a structurally important salt bridge to a lysine on M2) to provide a consistent measure between pLGICs. 

## 2. Materials and Methods

### 2.1. Cell Culture

HEK293 (human embryonic kidney) cells were grown at 37 °C in 7% CO_2_ in Dulbecco’s Modified Eagle’s Medium/Nutrient Mix F12 with GlutaMAX containing 10% fetal calf serum. Rat α4 and β2 nAChR genes with a L9’A mutation (Tapper et al. 2004) in pcDNA3.1 were modified by QuikChange site-directed mutagenesis and verified by nucleotide sequencing. For transfection, 5 μg of DNA in a α4:β2 1:2 ratio was incubated with 30 μg 25 kDa linear polyethyleneimine in DMEM/F12 for 10 min before being added to the HEK293 cells. Where relevant, 500 ng each of human NACHO (novel acetylcholine receptor chaperone) and human RIC-3 (resistance to inhibitors of cholinesterase-3) in pcDNA3.1 were also added [19,20].

### 2.2. FlexStation Analysis

As previously described [21], Flex buffer (10 mM HEPES, 115 mM NaCl, 1 mM KCl 1 mM CaCl_2_, 1 mM MgCl_2_, and 10 mM glucose, pH 7.4) containing blue fluorescent membrane potential dye (Molecular Devices) was added to cells 2 days post-transfection. After 45 min of incubation at 37 °C, the fluorescence responses to buffer or nicotine (added after 20 s) were measured every 2 s for 150 s on a FlexStation (Molecular Devices). Concentration–response curves were calculated using the equation $F=F_{min}+\frac{F_{max}-F_{min}}{1+10^{n_{H}(log\left({EC}_{50}-\left[A\right]\right))}}$ in GraphPad Prism v6.0, where F_max_ and F_min_ are the greatest and smallest recorded fluorescence values, [A] is the concentration of the agonist, and n_H_ is the Hill coefficient. 

### 2.3. Radioligand Binding

As previously described [22], a crude membrane preparation (which works well and uses less smaterial than a purified plasma membrane preparation) was prepared using cells harvested 2 days post-transfection, and this was incubated for 4 h at 4 °C in 50 mM Tris-HCl, pH 7.4 with [^3^H]epibatidine (62.2 Ci/mmol, PerkinElmer, Beaconsfield, UK). 300 μM nicotine was used to define nonspecific binding.

### 2.4. Protein Structure Prediction

AlphaFold2 [16,17] was used to predict the monomeric structures of full-length α4 and β2 sequences. These monomers were then aligned with their respective subunits in an experimentally determined truncated (α4)_2_ (β2)_3_ structure (PDB code 6CNJ) using PyMOL (The PyMOL Molecular Graphics System, Version 2.4.1, Schrödinger, LLC, New York, NY, USA), which was predicted to be the closed state. The open state was predicted using I-TASSER with default settings [23], using the α7 nAChR structure 7KOX as a structural template for both the monomers and then for the complete pentamer using PyMOL. 

## 3. Results

### 3.1. Nine Double-Alanine Mutations in the MA Helix Abolish Function

**Figure 3:**
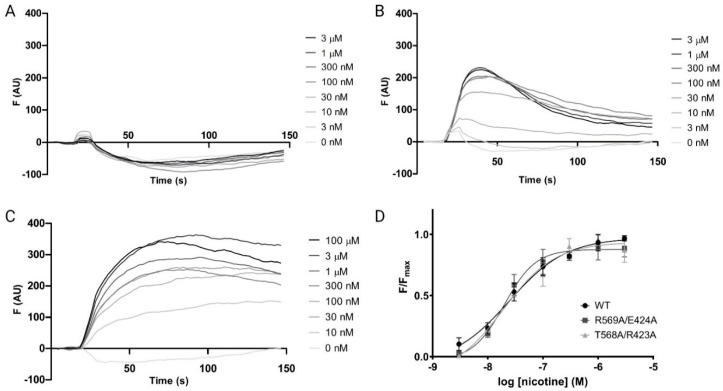
Characterization of α4β2 nAChRs in HEK293 cells. (**A**–**C**) Typical fluorescent responses (F, arbitrary units) to the addition of nicotine at 20 s to the mock transfected cells (**A**) or cells transfected with WT α4β2 nAChR (**B**) or WT α4β2 nAChR and chaperones RIC-3 and NACHO (**C**). (**D**) concentration–response curves from (**B**,**C**) and similar data (mean ± SEM, *n* ≥ 3).

Wild-type rat α4β2 nAChRs containing an L9’A mutation in the M2 helix of the α4 subunits (to enhance receptor responses to ligand [24,25], referred to as WT in the following text) showed concentration-dependent fluorescent responses to nicotine addition, revealing a pEC_50_ of 7.6 ± 0.12 (EC_50_ = 25.3 nM), similar to previous work [18], and a Hill slope (n_H_) of 0.8 ± 0.2 (Figure 3). Mutant receptors with similar EC_50_ values exhibited similar concentration–response curves (Figure 3D).

To explore the role of the MA helix in α4β2 nAChR function, we mutated each pair of MA residues (equivalent residues in the α4 and β2 MA helices; see Figure 2) to alanine, as well as selected prolines near the potential start of the MA helix. In the initial screening of 40 mutants, 30 showed WT-like function, and 10 had no response (Table 1). Simultaneous co-expression with the two chaperones RIC-3 and NACHO ([26,27], indicated by a ‘+’ appended to the mutant name) rescued the WT-like receptor function of one double mutant (I^MA^39A, indicating that the lack of response of this mutant in the initial assay was due to poor folding and/or export) but had no effect on the remaining nine.

### 3.2. Alanine Mutations Are Less Disruptive in the α4 Than in the β2 MA Helix

To determine the contributions of each subunit type at the ten mutation-sensitive MA positions, we characterized the corresponding receptors with MA mutations in only one of the subunit types (i.e., alanine mutant α4 subunits with WT β2 subunits and vice versa). 14 of these 20 single mutants showed WT-like function, and only 2 α4 and 4 β2 single mutants remained nonresponsive to the ligand (Table 2). This apparent dependence on β2 subunit residues over α4 subunit residues could be due to the different roles of the two subunits but is more likely due to the (α4)_2_(β2)_3_ stoichiometry of receptors used in this study, where any β2 mutation occurs three times in each pentamer but an α4 mutation only twice. The I^MA^39A double mutant required co-expression with the chaperones to show detectable function (Table 1), but both single mutants here showed WT-like function without requiring chaperones. While a mutation could change the assembly preference and final stoichiometry of the receptors, the wild-type (α4)_3_(β2)_2_ receptor has an EC_50_ about 30-fold smaller than the wild-type (α4)_2_(β2)_3_ receptor [28], and we observed no such shifts, indicating that the stoichiometry was likely unchanged by the mutations.

### 3.3. Two of the Nine Nonfunctional Mutant Receptors Are Expressed 

**Figure 4:**
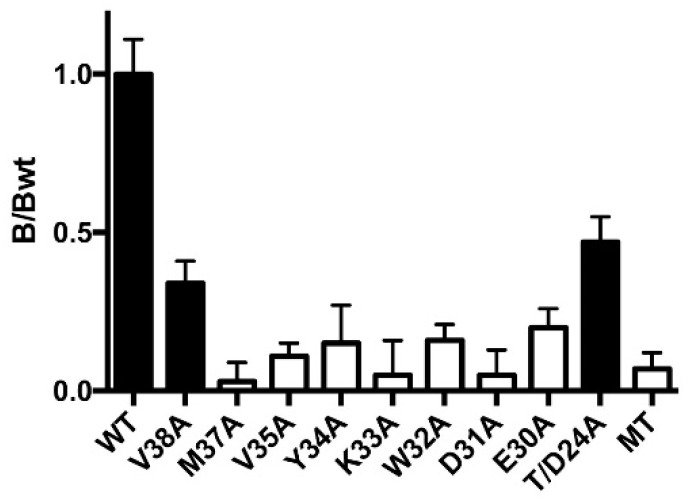
Specific binding of [^3^H]epibatidine relative to the WT in membranes from cells transfected with double-mutant nAChR subunit DNA, as indicated, and with RIC-3 and NACHO (MT indicates mock transfected cells). Black indicates significantly different to MT (*p* < 0.05). Data = mean ± SEM, n = 3.

To probe the expression of the nine MA double-mutant receptors that showed no response in the functional assay, we measured the [^3^H]epibatidine binding (Figure 4). While seven of the receptors showed no measurable binding, indicating that they are deficient in subunit folding and/or assembly, two (V^MA^38A+ and T/D^MA^24A+) showed significant levels of binding. This indicates that the lack of response in the functional assay for these two mutants is either due to the mutation preventing channel opening in response to ligand binding or to the receptors (which are assembled and capable of binding ligand) not having reached the plasma membrane.

### 3.4. Non-Alanine Mutations Reveal Required Characteristics of Key MA Helix Residues

To explore the residue requirements at the nine positions identified as crucial to receptor assembly, export, and/or function, we assessed the effects of a range of amino acid substitutions at each position (Table 3). Three positions (V^MA^38, V^MA^35, and W^MA^32) showed highly specific residue requirements, where even conservative substitutions abolished receptor function, and four tolerated only one of the assayed substitutions (E^MA^30, D^MA^31, K^MA^33, and Y^MA^34).

## 4. Discussion

The aim of this work was to explore the importance of the MA helix residues in receptor function by substituting them with alanine either in one subunit at a time (i.e., in two or three subunits of each pentamer) or in two subunits simultaneously (i.e., in all subunits of each pentamer). Alanine substitutions at 9 of the 40 positions tested abolished receptor responses, even on co-expression with chaperones RIC-3 and NACHO. Two of these non-responsive receptors showed ligand binding (V^MA^38A and T/D^MA^24A), indicating that those two residue pairs are involved either in receptor export to the plasma membrane or in allowing channel opening as a consequence of ligand binding. The remaining seven non-responsive mutant receptors showed no radioligand binding, indicating a disruption of receptor folding and/or assembly. Alanine substitutions at the remaining 30 positions tested had no measurable effect on receptor function.

### 4.1. Two Substitutions Abolished Detectable Ion Channel Function but Not Ligand Binding

**Figure 5:**
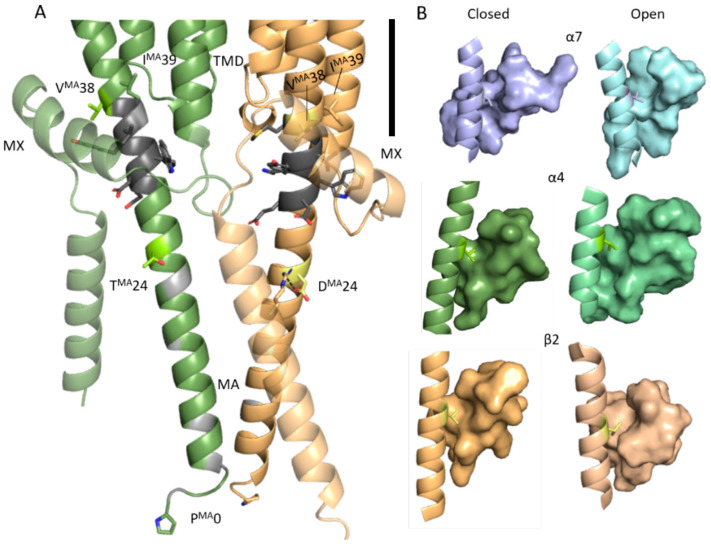
(**A**) MA helices of α4 (green) and β2 (yellow) in the closed α4β2 structure predicted by AlphaFold (which includes the full MA helix, showing MA helices going from P^MA^0 to I^MA^39 (both marked as sticks)). Black: positions where alanine mutations result in nonfunctional receptors incapable of ligand binding. Light colors: positions where alanine mutations result in nonfunctional receptors capable of ligand binding. Grey: positions where alanine mutations were not tested (most already alanines). The black line indicates the approximate location of the plasma membrane. (**B**) V^MA^38 and the MX helix in the closed and open conformations of α7, α4, and β2.

The T/D^MA^24A and V^MA^38A mutants showed no detectable function in the fluorescence assay (Table 1) but retained their ligand binding ability (Figure 4). V^MA^38 sits in a pocket defined by hydrophobic MX residues (α: F, V, P, and L; β: F, L, P, and L), and Thr substitution does not rescue function. This is reminiscent of the neuromuscular nAChR αV46 pin-into-socket gating hypothesis, which proposes a critical link between the ECD and the TMD is the side chain of αV46 tucked into a pocket formed by M2 [9,29,30]. Studies of this Val show its replacement by the isosteric Thr is deleterious, indicating the side-chain polarity is critical, and mutagenesis with unnatural amino acids shows the αV46 side chain methyl groups differentially affect gating, indicating they are in different environments [31]. We suggest that a V^MA^38 pin-into-socket link could play a role in gating by forming an essential link between the MA and MX helices, allowing a conformational change that opens the portals. In support of this, the α7 nAChR structure shows that the MX helix moves into the membrane on receptor opening (Figure 1D), and V^MA^38 moves with MX, thereby remaining in this pocket (Figure 5B) even as the MA helix unwinds and the C-terminal end of the M4 helix moves outwards on receptor activation (Figure 1D). In support of this hypothesis in the α7 nAChR, V^MA^38A reduces ACh-induced currents [11]. 

An alternative explanation is that V^MA^38A could affect plasma membrane expression, as receptors located on internal membranes but unable to reach the plasma membrane would also be detected in our assay. In support of this, in the α7 nAChR, V^MA^38A reduces receptor expression levels at the plasma membrane, in addition to its effect on currents [11]. However, we consider this unlikely here, as the subunit specific mutant receptors αV^MA^38 + βV^MA^38A and αV^MA^38A + βV^MA^38 both showed WT-like function, indicating that the receptors containing these mutant subunits were capable of reaching the plasma membrane. Thus, we suggest that the receptors are correctly assembled and targeted but are unable to undergo the conformational changes necessary for ion channel opening without the Val link. 

Individual alanine mutations at position ^MA^24 indicate that αT^MA^24, but not βD^MA^24, is crucial to receptor function (Table 2). This work used the high-sensitivity (α4)_2_(β2)_3_ receptor, so the βD^MA^24A mutation occurs in 3/5 subunits but has no detectable effect, while the αT^MA^24A mutation occurs in only 2/5 subunits but abolishes receptor responses in the functional assay, ruling out a simple dosage effect as the explanation for the relative importance of this residue between the two subunits. The receptors were assembled, as shown by the binding assay, and while these might have been retained in internal membranes, we consider this unlikely, as discussed above. We suggest a better explanation is that there is a critical functional interaction between αT^MA^24 and another residue in the ICD; in support of this hypothesis, a study of the α7 nAChR ICD found that E^MA^24 forms a salt bridge with an arginine in a small helix in the ICD named h3 [10], although its importance is unknown. Our structures of the nAChR α4 (P09483) and β2 (P12390) subunits do show a similar helix to h3 running parallel to the MA helix (perhaps unsurprising, as α7 was the template here), but no interactions are visible, perhaps indicating that our model is not sufficiently accurate in this region. It is also possible that there is an interaction with one of the chaperone proteins or with the lipid headgroups. Nevertheless, if there is an important interaction here, it is not widely conserved, as the (five-fold) E^MA^24A mutation in the 5-HT_3_A receptor has only minor effects on receptor function (a slight decrease in single-channel conductance) [32].

### 4.2. Other Alanine Substitutions in the MA Helix

Alanine substitutions at seven positions in a row (^MA^30-^MA^37) abolished both receptor function and ligand binding (Table 1 and Figure 4 and Figure 5), indicating that these mutant receptors were not properly folded and/or assembled within the cell. The first residue in this stretch, E^MA^30, has been shown to affect single-channel conductance levels, as mutating it to Arg effects a >two-fold change in single-channel conductance, and changes here also affect single-channel conductance in the 5-HT_3_A receptor (R4′ or R440) [12,13]. This stretch is also equivalent to the stretch in the α7 nAChR that unwinds and moves out on receptor opening (Figure 1D), so alterations here would be expected to alter function. However, the effects of mutations in this stretch are less deleterious in the α7 nAChR; alanine mutations in ^MA^32, ^MA^33, ^MA^34, and ^MA^38 (^MA^35 and ^MA^36 are already Ala) reduce but do not abolish expression and/or function [11]. 

Alanine substitutions at positions ^MA^16-^MA^32 in a mutant 5-HT_3_A receptor (where three arginines in the MA helix have already been replaced by a Gln, Asp, and Ala triad) all retained detectable channel function (though the W^MA^32A mutant showed only low levels of function) [32]. Thus, the data indicate that the exact composition of the MA helix is less crucial to function in the 5-HT_3_AR and α7nAChR than in the α4β2 nAChR. [33] proposed that MA movement is crucial for ion conductance in the 5-HT_3_A receptor at the level of residues ^MA^8 and ^MA^9 but not at the level of ^MA^15 and ^MA^16, though what the effects are around ^MA^24 and further C-terminal of that, remain to be determined.

### 4.3. Other Non-Alanine Substitutions in the MA Helix

Lysine substitutions at positions ^MA^30 and ^MA^31 in the α4β2 nAChR both abolished detectable receptor response to ligand. While not precisely comparable, we note that ^MA^30 is an arginine in the 5-HT_3_A receptor, and the D^MA^31R mutation does not reduce receptor function [32], demonstrating that positively charged residues in the MA have different effects at the same positions in these two receptors. In fact, the negative charge is indicated to be specifically important at both these positions by the fact that D^MA^31E and E^MA^30D are both tolerated substitutions but D^MA^31N or E^MA^30Q (Table 3) are not.

## 5. Conclusions

In this work, we have shown that a region of the α4β2 nAChR MA helix (^MA^30-^MA^37) plays a role in receptor assembly while two residues (^MA^38 and ^MA^24) are involved in receptor function. The latter contrasts with data from the α7 nAChR and 5-HT_3_AR, where equivalent mutations do not ablate function, suggesting that the specific roles of the MA helix residues can vary between different cation-selective pLGICs.

## Figures and Tables

**Table 1:** Parameters from the MA Ala mutants. Data = mean ± SEM, *n* = 3–5, NR = non-responsive.

Position	Mutation (α4/β2)	pEC_50_ (M)	EC_50_ (nM)	n_H_
WT		7.60 ± 0.12	25.3	0.8 ± 0.2
WT+		7.94 ± 0.12	11.4	0.6 ± 0.1
I^MA^39A+	I601A/I456A	8.11 ± 0.12	8	1.3 ± 0.4
V^MA^38A+	V600A/V455A	NR		
M^MA^37A+	M599A/M454A	NR		
V^MA^35A+	V597A/V452A	NR		
Y^MA^34A+	Y596A/Y451A	NR		
K^MA^33A+	K595A/K450A	NR		
W^MA^32A+	W594A/W449A	NR		
D^MA^31A+	D593A/D448A	NR		
E^MA^30A+	E592A/E447A	NR		
K/R^MA^29A	K591A/R446A	7.62 ± 0.06	24	1.1 ± 0.2
V^MA^28A	V590A/V445A	7.62 ± 0.06	24	1.5 ± 0.3
S^MA^27A	S589A/S444A	7.47 ± 0.06	34	1.2 ± 0.2
F/Q^MA^26A	F588A/Q443A	7.83 ± 0.06	14.7	1.2 ± 0.2
D^MA^25A	D587A/D442A	7.79 ± 0.09	16.2	1.4 ± 0.4
T/D^MA^24A+	T586A/D441A	NR		
D^MA^23A	D585A/D440A	7.89 ± 0.08	12.8	1.1 ± 0.3
E^MA^22A	E584A/E439A	7.59 ± 0.05	25.3	2.1 ± 0.5
WT/S^MA^21A	WT/S438A	7.36 ± 0.03	43.2	1.6 ± 0.2
K/R^MA^20A	K582A/R437A	7.76 ± 0.08	17.2	1.3 ± 0.2
L/M^MA^19A	L581A/M436A	7.63 ± 0.09	23.5	1.5 ± 0.4
H^MA^18A	H580A/H435A	7.70 ± 0.19	20.1	1.9 ± 1.3
D^MA^17A	D579A/D434A	7.61 ± 0.13	24.7	2.6 ± 2.1
I^MA^15A	I577A/I432A	7.71 ± 0.10	19.6	1.3 ± 0.4
Y/F^MA^14A	Y576A/F431A	7.99 ± 0.05	10.2	1.2 ± 0.1
Q/R^MA^13A	Q575A/R430A	7.73 ± 0.13	18.6	1.1 ± 0.3
V^MA^12A	V574A/V429A	7.55 ± 0.14	28.0	1.2 ± 0.4
G^MA^11A	G573A/G428A	7.23 ± 0.27	58.0	0.8 ± 0.5
E/D^MA^10A	E572A/D427A	7.55 ± 0.11	28.5	1.3 ± 0.3
V^MA^9A	V571A/V426A	7.79 ± 0.10	16.4	1.2 ± 0.2
R/E^MA^7A	R569A/E424A	7.73 ± 0.16	18.5	1.6 ± 0.8
T/R^MA^6A	T568A/R423A	7.72 ± 0.24	19.3	0.9 ± 0.4
L^MA^5A	L567A/L422A	7.63 ± 0.10	23.2	0.9 ± 0.1
WT/G^MA^4A	WT/G421A	7.91 ± 0.10	12.1	1.7 ± 0.5
P/C^MA^3A	P565A/C420A	7.79 ± 0.06	16.1	1.1 ± 0.2
S^MA^2A	S564A/S419A	7.61 ± 0.08	24.3	1.2 ± 0.3
P^MA^0A	P562A/P417A	7.67 ± 0.12	21.6	1.0 ± 0.2
	P558A/WT	7.69 ± 0.14	20.3	1.4 ± 0.5
	P557A/WT	7.75 ± 0.18	17.6	1.1 ± 0.4
	WT/P411A	7.78 ± 0.07	16.6	1.1 ± 0.2
	WT/P406A	7.67 ± 0.08	21.4	1.0 ± 0.2

**Table 2:** Parameters from the MA single subunit Ala mutants. Data = mean ± SEM, *n* = 3–5, NR = non-responsive.

	Mutant α4 WT β2			WT α4 Mutant β2		
Position	pEC_50_ (M)	EC_50_ (nM)	n_H_	pEC_50_ (M)	EC_50_ (nM)	n_H_
WT	7.60 ± 0.12	25.3	0.8 ± 0.2	7.60 ± 0.12	25.3	0.8 ± 0.2
WT+	7.94 ± 0.12	11.4	0.6 ± 0.1	7.94 ± 0.12	11.4	0.6 ± 0.1
I^MA^39A	7.81 ± 0.06	15	1.9 ± 0.4	7.41 ± 0.08	39	1.0 ± 0.2
V^MA^38A+	7.20 ± 0.24	63.3	0.8 ± 0.4	7.26 ± 0.11	55	1.2 ± 0.3
M^MA^37A+	7.53 ± 0.06	30	1.0 ± 0.1	NR		
V^MA^35A+	7.32 ± 0.10	48.1	1.5 ± 0.4	7.67 ± 0.17	21	1.3 ± 0.7
Y^MA^34A+	7.46 ± 0.08	34.8	1.2 ± 0.2	7.46 ± 0.08	34	1.3 ± 0.3
K^MA^33A+	7.63 ± 0.17	23.2	0.7 ± 0.2	NR		
W^MA^32A+	NR			NR		
D^MA^31A+	7.53 ± 0.08	29.7	1.3 ± 0.3	NR		
E^MA^30A+	7.38 ± 0.08	41.9	0.8 ± 0.1	7.78 ± 0.07	17	1.5 ± 0.4
T/D^MA^24A+	NR			7.59 ± 0.12	26.0	1.0 ± 0.2

**Table 3:** Parameters from receptors mutated at sensitive MA residues. Data = mean ± SEM, *n* = 3–5, NR = non-responsive.

Position	pEC_50_ (M)	EC_50_ (nM)	n_H_
WT	7.60 ± 0.12	25.3	0.8 ± 0.2
WT+	7.94 ± 0.12	11.4	0.6 ± 0.1
V^MA^38I+	NR		
V^MA^38T+	NR		
M^MA^37K+	NR		
V^MA^35I+	NR		
V^MA^35T+	NR		
Y^MA^34F	7.21 ± 0.16	61.2	0.88 ± 0.3
Y^MA^34S+	NR		
Y^MA^34L+	NR		
Y^MA^34Q+	NR		
K^MA^33E	7.02 ± 0.06	95.4	1.1 ± 0.2
K^MA^33Q+	NR		
K^MA^33M+	NR		
W^MA^32F+	NR		
W^MA^32Y+	NR		
D^MA^31E	7.43 ± 0.09	37.4	1.2 ± 0.3
D^MA^31K+	NR		
D^MA^31L+	NR		
D^MA^31N+	NR		
E^MA^30D	7.48 ± 0.04	32.9	1.6 ± 0.2
E^MA^30K+	NR		
E^MA^30L+	NR		
E^MA^30Q+	NR		
αT^MA^24D	7.50 ± 0.05	31.4	1.6 ± 0.3
αT^MA^24E	7.14 ± 0.28	72.4	1.2 ± 0.8
αT^MA^24K	7.41 ± 0.10	39.4	1.4 ± 0.4
αT^MA^24S	7.80 ± 0.04	15.9	1.6 ± 0.2
αT^MA^24V+	NR		
βD^MA^24T+	7.76 ± 0.10	17.3	1.1 ± 0.2

## Data Availability

Data is contained within the article.
